# Teleophthalmology and Teleglaucoma in Clinical Practice: Attitudes of Ophthalmologists in Bulgaria

**DOI:** 10.3390/healthcare14121696

**Published:** 2026-06-13

**Authors:** Stanka Uzunova, Rumyana Stoyanova, Marin Atanassov, Angel Atanasov, Kristina Kilova

**Affiliations:** 1 Department of Health Management and Health Economics, Faculty of Public Health, Medical University of Plovdiv, 4002 Plovdiv, Bulgaria; suzunova@doctor.com; 2 Department of Ophthalmology, Faculty of Medicine, Medical University of Plovdiv, 4002 Plovdiv, Bulgaria; marin.atanassov@mu-plovdiv.bg (M.A.); angel.atanasov@mu-plovdiv.bg (A.A.); 3 Department of Medical Informatics, Biostatistics and E-Learning, Faculty of Public Health, Medical University of Plovdiv, 4002 Plovdiv, Bulgaria; kristina.kilova@mu-plovdiv.bg

**Keywords:** glaucoma, ophthalmologists, telemedicine, telemonitoring, teleophthalmology, virtual clinics

## Abstract

**Background:** Over the past two decades, teleophthalmology has become an effective approach for glaucoma screening and follow-up, with its adoption markedly accelerated by the COVID-19 pandemic. **Objectives:** The aim of the present study was to explore and analyze the attitudes of ophthalmologists in Bulgaria toward the application of teleglaucoma, digital communication, and artificial intelligence in clinical practice. **Methods:** A cross-sectional survey study was conducted among 113 ophthalmologists between September 2024 and March 2025, representing 10.5% of all licensed ophthalmologists in Bulgaria (n = 1074). **Results:** Age, professional experience, and specialization influenced the level of involvement in managing glaucoma patients. The level of awareness regarding the term ‘teleophthalmology’ was higher among respondents with a specialization in ophthalmology and those holding a doctoral degree (*p* = 0.001). Among the ophthalmologists surveyed, 35.4% (n = 40) provided teleophthalmology services, while an additional 19.5% (n = 22) reported no prior provision of such services but planned to do so in the future. The most preferred method for conducting teleophthalmology consultations was telephone communication (n = 27; 67.5%), followed by communication via Skype, Viber, or Messenger (n = 23; 57.5%). Physicians with longer professional experience more frequently conducted remote consultations with patients they already knew (*p* = 0.006). A substantial proportion of respondents (85.0%, n = 96) expressed willingness to participate in training related to contemporary trends and the provision of remote medical services. More than half of respondents expressed positive attitudes toward the use of artificial intelligence in ophthalmology, although practical implementation remained limited. **Conclusions:** The present study outlined the current landscape of attitudes among ophthalmologists in Bulgaria toward teleglaucoma, digital communication, and the use of artificial intelligence in clinical practice. The findings indicated a moderately positive yet cautious stance—remote services were perceived primarily as complementary tools, particularly for the follow-up of previously known patients and for real-time collaboration between specialists.

## 1. Introduction

The increased life expectancy in contemporary society is leading to a growing need for ophthalmological care, particularly among the aging population. Eye diseases are among the leading causes of disability and reduced quality of life, and without adequate prevention and treatment, the number of people with visual impairments continues to increase. Globally, the number of blind individuals increased from 44 million in 2000 to 76 million in 2020, clearly illustrating the scale of the problem. In Bulgaria, in 2020, approximately 1.3 million people were living with vision loss, around 20,000 of whom were completely blind [1].

Alongside the increasing prevalence of eye diseases, access to specialized ophthalmological care remains a significant challenge. According to data from the National Statistical Institute, as of 31 December 2024, a total of 30,149 physicians were practicing in Bulgaria, of whom 1074 were ophthalmologists, representing only 3.56% of all medical specialists [2]. At the same time, their distribution is highly uneven, with a marked concentration in large urban centers and a serious shortage in less densely populated regions.

Similar trends are observed at the international level. In the United States, despite a relatively high number of ophthalmologists, their national density is declining, while the proportion of aging specialists is increasing. Projections indicate a significant shortage of ophthalmologists relative to expected demand by 2025 [3]. Globally, inequalities are even more pronounced—in India, one ophthalmologist serves approximately 400,000 people, and in some regions of Africa, one serves up to 1 million people [4]. By comparison, in Europe this ratio is approximately 1:10,000, clearly highlighting the disparities in access to eye care.

Data from 2015 shows that approximately 232,866 ophthalmologists were practicing across 194 countries, with their density being strongly dependent on the respective country’s level of economic development—ranging from an average of 3.7 per million population in low-income countries to 76.2 in high-income countries [5]. In response to these challenges, the World Health Organization, in its document ‘Universal Eye Health: A Global Action Plan 2014–2019’, recommends systematic monitoring and reporting of the number of eye care specialists as a key indicator of progress in ensuring access to eye health services [6].

In this context, internet-based technologies for delivering eye care—collectively referred to as teleophthalmology—are regarded as a sustainable solution for overcoming geographic and workforce limitations [7]. Teleophthalmology is a branch of telemedicine that uses digital medical equipment and telecommunications technologies to provide access to diagnosis, treatment, prevention, monitoring, and education in the field of ophthalmology [8]. It enables screening, remote consultations, and patient follow-up, particularly in remote and underserved regions, while simultaneously optimizing specialists’ time and resources.

Over the past two decades, teleophthalmology has developed dynamically across a number of advanced healthcare systems in Europe, North America, and other regions worldwide, with virtual glaucoma clinics (VGCs) gradually emerging as an effective model for monitoring patients with glaucoma. This process was significantly accelerated by the COVID-19 pandemic, declared in March 2020, which restricted access to routine healthcare services and underscored the need to integrate telemedicine into clinical practice at an international level [9,10]. Data indicates that during the pandemic, in countries where telemedicine services had already been implemented, 77.4% of eye care providers delivered telehealth services, even though most of them had not used this model prior to the emergence of COVID-19 [11].

In this context, virtual glaucoma clinics (VGCs) have evolved as a structured form of teleglaucoma, in which the process of clinical measurements is separated from clinical decision-making. Data is collected by trained personnel and subsequently assessed remotely by ophthalmologists or glaucoma specialists [12,13]. The implementation of this model leads to reduced time spent in the clinic, increased capacity of ophthalmological services, and more efficient management of patient flow [14]. National studies in the United Kingdom show that half of the leading eye clinics are already operating VGCs, while a substantial proportion of the remaining clinics are planning their introduction [15,16].

Despite the growing international adoption of teleglaucoma and virtual models of care, data on the attitudes and experiences of clinicians in Bulgaria remain limited. This is particularly significant in light of the uneven territorial distribution and the relative shortage of ophthalmologists in the country, which complicates the provision of timely access to specialized eye care. In this context, understanding ophthalmologists’ perspectives on innovative approaches such as teleglaucoma is a key factor for their successful implementation and sustainable development in clinical practice—especially within a healthcare system where telemedicine has not yet been widely integrated.

The aim of the present study was to explore and analyze the attitudes of ophthalmologists in Bulgaria toward the application of teleglaucoma, digital communication, and artificial intelligence in clinical practice.

## 2. Materials and Methods

A cross-sectional survey study was conducted among 113 ophthalmologists between September 2024 and March 2025, representing 10.5% of all licensed ophthalmologists in Bulgaria (n = 1074), thus providing substantial national coverage for an exploratory survey study. The survey was distributed by the Bulgarian Society of Ophthalmology through professional communication channels, including electronic mailing lists, and was completed voluntarily and anonymously. Because the survey was distributed through professional mailing lists and open society channels, the exact response rate could not be determined. A formal sample size calculation was not performed due to the exploratory nature of the study and the voluntary participation design.

The survey instrument was developed by the authors based on a review of the relevant literature on teleophthalmology, teleglaucoma, and digital health implementation in ophthalmic practice. Prior to dissemination, the questionnaire underwent face and content validity assessment by the research team, consisting of ophthalmologists with clinical and academic experience in glaucoma care, in order to ensure clarity, relevance, and comprehensibility of the survey items. The total number of questions was 29. The first set of questions in the survey was demographic; the second set covered the main subject matter. A portion of the questions employed R. Likert’s gradation scale, with possible choices including: ‘yes’, ‘somewhat yes’, ‘undecided’, ‘somewhat no’, and ‘no’. In another section of the questionnaire, multiple answers were allowed. The survey also included a separate section for respondents who had already offered a telemedicine service.

The questionnaires were primarily completed online using a Google Form specifically designed for survey research. All mandatory fields were completed in full, resulting in no responses being excluded from the analysis.

The English version of the questionnaire is provided as Supplementary Material S1.

The study complied with established standards and adhered to the requirements of the Declaration of Helsinki on ethical principles for medical research and the principles of Good Clinical Practice. It had been approved by the Research Ethics Committee at the Medical University of Plovdiv (Opinion P-KHE-13/14.04.2025).

### Statistical Analysis

The data was analyzed using descriptive statistics, along with parametric and non-parametric tests to verify hypotheses. Central tendency measures were reported as mean (M) and standard deviation (SD). Qualitative data was presented as absolute frequencies and relative percentages. Associations between categorical variables were examined using Pearson’s Chi-Square test (χ^2^) for multi-dimensional tables and Fisher’s Exact Test for 2 × 2 tables. Analysis of variance (One-way ANOVA) was applied to compare normally distributed quantitative indicators across more than two groups. A *p*-value of less than 0.05 was considered statistically significant for rejecting the null hypothesis.

The data was statistically processed using the software products SPSS 23 and MS Excel 2016. Open-ended responses were reviewed qualitatively by members of the research team and grouped into broad descriptive categories reflecting common themes expressed by participants. The comments were summarized descriptively to provide contextual insights into the quantitative findings, without formal qualitative coding or thematic analysis.

## 3. Results

The survey was completed by 113 ophthalmologists, of whom 82 were women (72.6%) and 31 were men (27.4%). The mean age was 47.07 ± 14.93 years, indicating that most respondents were of active working age. Fifteen percent (n = 17) held a doctoral degree, while 14.2% (n = 16) did not have a specialization in ophthalmology. More than half of the physicians had over 10 years of professional experience, and approximately half had more than 20 years of experience (Table 1).

In view of their professional interests, the participants were asked whether they worked with patients with glaucoma. The results showed that 97.3% (n = 110) had experience with this condition, and for 26.5% (n = 30), glaucoma was among their primary areas of interest, highlighting its significance in clinical practice.

Non-parametric analysis showed that age, length of professional experience, and specialization influenced the level of involvement in managing patients with glaucoma. Physicians who did not work with glaucoma patients had a mean age of 36.0 ± 8.718 years, whereas those for whom glaucoma represented a primary professional interest had a mean age of 54.5 ± 12.462 years (*p* = 0.004). The mean length of professional experience among those actively engaged in managing the condition was 26.1 ± 12.011 years, compared to 7.33 ± 5.859 years among those who did not work with it (*p* = 0.003). Moreover, 30.9% (n = 30) of ophthalmology specialists identified glaucoma as a primary area of interest, while none of the non-specialized physicians did so (*p* = 0.002).

The first question related to ophthalmologists’ attitudes toward providing services remotely was introductory (control in nature) and aimed to assess their familiarity with the term ‘teleophthalmology’. The majority of respondents provided a positive response (n = 107; 94.7%). Only two participants (1.8%) indicated ‘cannot assess’, while four respondents (3.5%) answered ‘somewhat no’.

The survey data showed that the level of awareness regarding the term ‘teleophthalmology‘ was significantly higher among respondents with a specialization in ophthalmology and those holding a doctoral degree. Among the ophthalmologists surveyed, 64.9% (n = 63) clearly stated that they knew what the term encompassed, whereas this proportion was 37.5% (n = 6) among physicians without a specialization. Statistical analysis demonstrated a significant association between having a specialization in ophthalmology and familiarity with the topic (*p* = 0.001).

A similar trend was observed with respect to educational level. Among participants holding a doctoral degree, 94.1% (n = 16) stated that they were familiar with the term, whereas this applied to only 55.2% (n = 53) of those holding a master’s degree. A statistically significant association was also identified in this case (*p* = 0.026).

Depending on the response to the next question—whether they previously provided such services—the survey proceeded in different ways through a dedicated section.

### 3.1. Experience of Ophthalmologists with Teleophthalmology Services

Among the ophthalmologists surveyed, 35.4% (n = 40) provided teleophthalmology services, while an additional 19.5% (n = 22) reported no prior provision of such services but planned to do so in the future. A small proportion of respondents (n = 10; 8.8%) reported that they were not familiar with this type of service. A negative response was given by 36.3% (n = 41), suggesting that opinions ‘for‘ and ‘against‘ were almost evenly distributed among the respondents.

**Figure 1:**
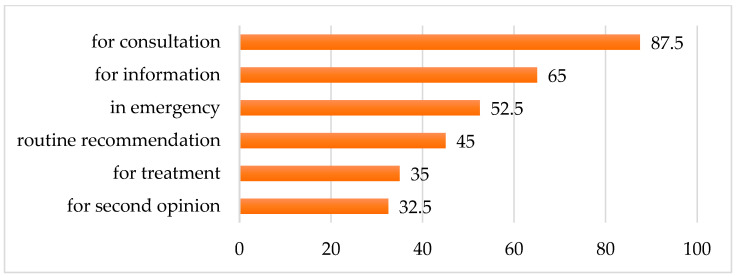
Results on what purposes teleophthalmology services were provided (in %). Multiple responses were allowed. Percentages were calculated only for respondents who reported prior experience with teleophthalmology services (n = 40).

The ophthalmologists surveyed provided remote medical services primarily for consultations (n = 35; 87.5%). A substantial proportion also used remote services to provide information (n = 26; 65%) or to manage emergency cases (n = 21; 52.5%). Approximately 45% (n = 18) of respondents indicated that they offered lifestyle or management recommendations remotely, while 35% (n = 14) used remote communication for treatment purposes. The smallest proportion of physicians provided second opinions through remote services (n = 13; 32.5%). For this question, respondents were allowed to select more than one answer. The results are presented in Figure 1.

**Figure 2:**
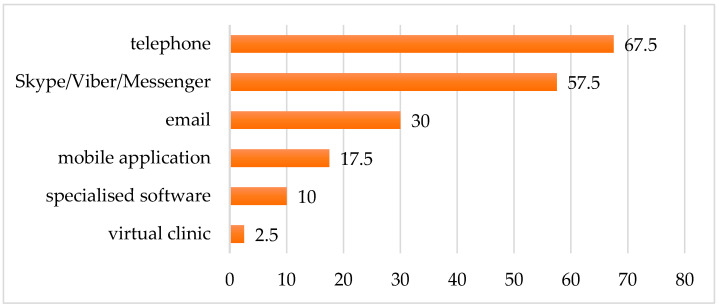
How the teleophthalmology service was delivered (in %). Multiple responses were allowed. Percentages were calculated only for respondents who reported prior experience with teleophthalmology services (n = 40).

Respondents used various methods to conduct teleophthalmology consultations. The most preferred method was telephone communication (n = 27; 67.5%), followed by communication via Skype, Viber, or Messenger (n = 23; 57.5%). Approximately 30% (n = 12) consulted their patients via email, while 17.5% (n = 7) used mobile applications. Specialized software was applied by 10% (n = 4) of the respondents, and virtual clinics were used in isolated cases (n = 1; 2.5%). The data showed that most physicians relied on widely accessible and familiar communication tools, whereas specialized telemedicine platforms were still used to a limited extent. The responses are presented in Figure 2.

Levels of satisfaction with the teleophthalmology consultations conducted varied. The largest proportion of respondents rated their experience as good (43.6%, n = 17), while 28.2% (n = 11) described their satisfaction as excellent. A total of 17.9% (n = 7) considered the consultations satisfactory, whereas 10.3% (n = 4) stated that they were not satisfied. There were no responses indicating that they would not offer this type of service again.

In response to the question of whether they knew the patient with whom the teleophthalmology consultation was conducted, the results showed that 32.5% (n = 13) of ophthalmologists answered definitively ‘yes’. A total of 55.0% (n = 22) indicated that some of the patients were not previously known to them, while 12.5% (n = 5) stated that all individuals consulted in this manner were unknown to them. According to the data obtained, remote consultations were conducted both with existing patients and with new ones.

A statistically significant difference was identified according to length of professional experience (*p* = 0.006). Physicians with longer professional experience more frequently conducted remote consultations with patients they already knew, likely due to established professional relationships and a developed foundation of trust. Conversely, younger specialists more often worked with new or previously unknown patients, which was consistent with their shorter clinical experience.

More than half of the surveyed ophthalmologists (55%, n = 22) conducted remote consultations with patients suspected of having glaucoma or with an already established diagnosis.

Subsequently, the survey proceeded in an identical manner for those who responded positively or negatively to the second question.

### 3.2. Attitudes Toward the Provision of Medical Services Remotely

#### 3.2.1. Attitudes Toward Remote Consultations for Patients Suspected of Having Glaucoma or Belonging to Risk Groups

The results showed a relatively balanced distribution of attitudes regarding the provision of remote consultations for patients suspected of having glaucoma or belonging to risk groups. A positive attitude was expressed by 53.9% of respondents (n = 61), while 38.9% (n = 34) demonstrated a negative attitude. A small proportion of participants were unable to provide a definitive answer (n = 8; 7.1%) (see Table 2; Q1).

The analysis of attitudes toward providing remote treatment recommendations for patients with established glaucoma showed an almost even distribution. Overall, 46.0% of physicians (n = 52) were rather opposed or strongly opposed to delivering such services remotely, while 45.1% (n = 51) were inclined to offer remote treatment recommendations. The proportion of respondents who were undecided was 8.8% (n = 10) (see Table 2; Q2).

#### 3.2.2. Influence of Prior Knowledge of the Patient on Remote Consultation

The analysis of attitudes toward remotely consulting previously unknown patients revealed a predominantly negative stance. More than half of the ophthalmologists surveyed (59.3%; n = 67) stated that they would not provide remote consultations to patients they did not know, while 35.4% (n = 40) were willing to accept such practice. A small proportion of respondents (5.3%; n = 6) were unable to give a definitive answer (see Table 2; Q3).

In contrast, attitudes were significantly more positive when the patient was already known to the physician. More than two-thirds of respondents (68.2%; n = 77) stated that they would conduct remote consultations in such cases, while 30.1% (n = 34) responded negatively (see Table 2; Q4).

#### 3.2.3. Types of Remote Services Provided to Patients with Glaucoma

The analysis of attitudes regarding remote follow-up of patients with established glaucoma for routine check-ups showed that opinions were relatively balanced. Nearly half of the physicians (47.8%; n = 54) were inclined to use remote follow-up, while 43.4% (n = 49) expressed skepticism. The proportion of undecided respondents was 8.8% (n = 10) (see Table 2; Q5).

In the context of remote consultations for patients with glaucoma in emergency situations, a clearly expressed caution was observed. More than half of the physicians surveyed (56.6%; n = 64) were inclined to decline such practice, while 34.5% (n = 39) stated that they would offer a remote consultation if necessary (see Table 2; Q6).

**Figure 3:**
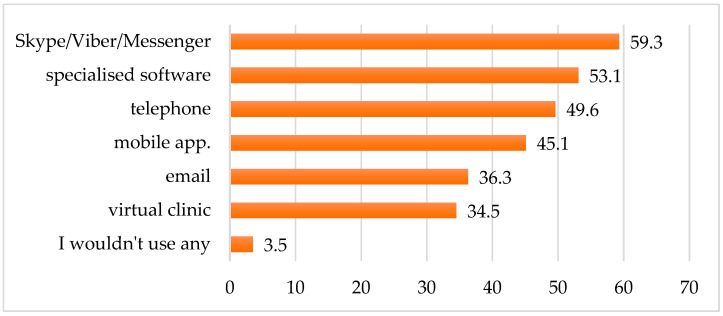
Preferred type of communication by ophthalmologists (in %). Multiple responses were allowed. Percentages were calculated based on the total number of respondents (n = 113).

Respondents were asked about their preferred type of communication when providing remote services. The total number of responses was 318, as participants were allowed to select more than one option. The data showed that widely accessible communication channels such as Skype, Viber, and Messenger were the most preferred (n = 67; 59.3%). A high proportion of physicians also indicated that they would use specialized software (n = 60; 53.1%), telephone calls (n = 56; 49.6%), or mobile applications (n = 51; 45.1%). Virtual clinics and email were less preferred. Only 3.5% of respondents categorically refused to use telemedicine solutions. The data are presented in Figure 3.

#### 3.2.4. Training and Remote Consultations with Fellow Ophthalmologists

A substantial proportion of the surveyed physicians (85.0%, n = 96) expressed willingness to participate in training related to contemporary trends and the provision of remote medical services. Only 9.7% (n = 11) stated that they would not participate (see Table 2; Q7).

Regarding the possibility of real-time remote consultation with another ophthalmologist while the patient was present in the office, strong support was observed—80.6% (n = 91) of physicians were willing to use such a model. Those expressing skepticism accounted for 9.7% (n = 11), as did the undecided respondents (9.7%, n = 11) (see Table 2; Q8).

A statistically significant association was identified between age and attitudes toward this type of consultation (*p* = 0.025), with the highest level of willingness observed among physicians aged 46–47 years.

#### 3.2.5. Virtual Clinic and Consultations with Other Specialists

With regard to the possibility of offering a real-time remote consultation with another specialist, 59.2% (n = 67) of the surveyed ophthalmologists expressed a positive opinion (‘somewhat yes’ and ‘yes’). A neutral position was reported by 26.5% (n = 30) of respondents, while 14.1% (n = 16) demonstrated a negative attitude (see Table 2; Q9).

A similar trend was observed in response to the question regarding conducting a consultation or secondary examination of a patient with glaucoma through a virtual clinic. A positive attitude was expressed by 60.2% (n = 68) of respondents. A neutral position was reported by 15.0% (n = 17), while 24.8% (n = 28) expressed a rather negative or negative opinion (see Table 2; Q10).

An additional question revealed that physicians recognized the value of various digital tools for improving communication with patients. The highest proportion of respondents (54.9%, n = 62) indicated that the ability to share visual information, such as images and examination results, would contribute to better diagnosis and follow-up of patients’ conditions. The virtual clinic was also evaluated as a useful tool by 40.7% (n = 46) of respondents, as a means of facilitating consultations and improving patient access.

The total number of responses indicated was 287, demonstrating that a substantial proportion of physicians supported the introduction of more than one digital solution. Telephone and video calls were also regarded as useful; however, priority was given to structured online platforms and to the possibilities for exchanging medical information.

A skeptical attitude toward digital solutions was expressed by 10.6% (n = 12) of respondents, who stated that such tools would not improve their work. Two participants selected the response ‘other’, with one indicating that telephone calls would limit their personal time and the other stating that they already used all of the listed options.

**Figure 4:**
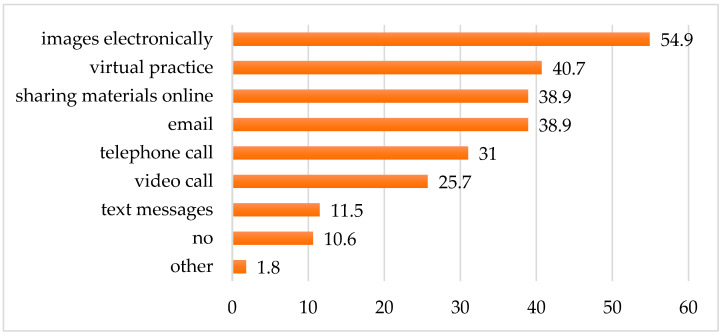
Ophthalmologists’ opinions regarding the improvement of physician–patient communication (in %). Multiple responses were allowed. Percentages were calculated based on the total number of respondents (n = 113).

The data are presented in Figure 4.

**Figure 5:**
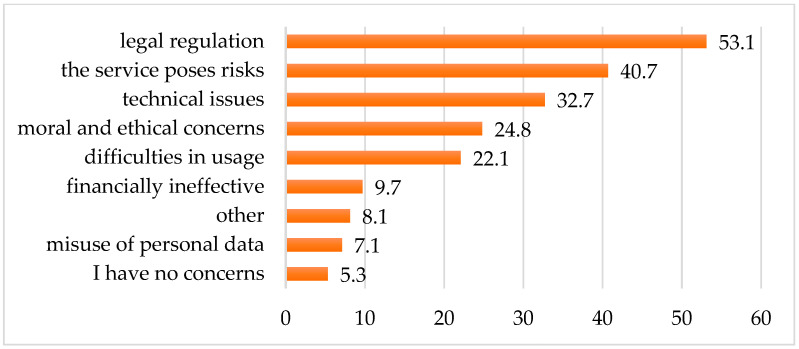
Ophthalmologists’ concerns regarding the use of telemedicine services (in %). Multiple responses were allowed. Percentages were calculated based on the total number of respondents (n = 113).

Ophthalmologists were also asked about their concerns regarding the use of remote medical services. The total number of responses received was 229. The surveyed physicians expressed considerable concerns about providing medical services at a distance. The main challenges identified were the lack of legal regulation (53.1%), risks of inaccurate diagnosis (40.7%), technical problems (32.7%), and moral and ethical considerations (24.8%). In addition, 22.1% believed that patients might experience difficulties in using telemedicine platforms, and 7.1% pointed to the risk of misuse of personal data. Although some physicians supported the idea of remote consultations, significant reservations remained. The data are presented in Figure 5.

#### 3.2.6. Digital Communication, Awareness, and Adherence to Therapy in Glaucoma

The majority of respondents (79.6%, n = 90) believed that adherence to therapy among patients with glaucoma would improve if opportunities for physician–patient communication were expanded. (see Table 2; Q11).

An even higher proportion of positive responses was observed regarding the impact of increased patient awareness of the disease, including through mobile applications. The total share of positive responses amounted to 87.6% (n = 99), with half of the respondents (50.4%, n = 57) fully supporting this statement. Only 12.4% (n = 14) expressed a neutral or negative opinion (see Table 2; Q12).

A statistically significant association was identified with respect to sex on this issue (*p* = 0.033). A significantly higher proportion of women (57.3%, n = 47) provided a positive response and believed that increased patient awareness would improve adherence to therapy, compared to men (32.3%, n = 10).

Regarding the usefulness of mobile applications in ophthalmology, 81.5% (n = 92) of respondents evaluated them as useful. A neutral attitude was expressed by 12.4% (n = 14), while only 6.2% (n = 6) of participants expressed a negative opinion (see Table 2; Q13).

Pearson’s Chi-Square test demonstrated a significant association between the respondents’ level of education and their attitudes toward mobile applications in ophthalmology. Participants holding a doctoral degree showed the highest level of support, with 100% (n = 17) considering mobile applications to be useful (*p* = 0.011). Among the remaining participants, more uncertain responses or reservations were observed.

#### 3.2.7. Attitudes Toward the Use of Artificial Intelligence (AI) in Ophthalmology

More than half of the ophthalmologists perceived potential in AI technology and responded positively (51.3%, n = 58). Nevertheless, over one quarter (27.4%, n = 31) indicated that they were unable to assess its impact, likely due to a lack of sufficient experience with real-world AI tools. A total of 14.2% (n = 16) believed that AI would rather not be helpful, while 7.1% (n = 8) were categorically opposed (see Table 2; Q14). Although more than half of the respondents supported the use of AI to improve physician–patient interaction, a considerable proportion remained uncertain or skeptical.

Regarding the use of AI (e.g., ChatGPT) in daily professional practice, 49.5% (n = 56) of respondents stated that they would be willing to use it if given the opportunity. A neutral position was expressed by 25.7% (n = 29), while 24.7% (n = 28) demonstrated a rather negative attitude (see Table 2; Q15).

Responses to the subsequent question indicated that AI was used to a limited extent among physicians. Approximately one quarter of respondents (23.9%, n = 27) used AI for work-related questions, while an additional 6.2% (n = 7) reported using it regularly. At the same time, the results showed a high level of non-use or lack of interest—53.1% (n = 60) of physicians never used AI, and a small proportion (4.4%, n = 5) stated that they would not use it. A notable proportion of respondents were also unfamiliar with AI tools (12.4%, n = 14). Although AI began to enter the field of medicine, most physicians did not actively integrate it into their work.

The results of the analysis showed that the differences were statistically significant and that length of professional experience influenced attitudes toward the use of AI in professional practice (*p* = 0.018). The mean years of experience were the highest among respondents who were unfamiliar with the technology (27.21 ± 9.721 years) and the lowest among those who used AI regularly (12.29 ± 10.275 years). Younger physicians were more likely to use AI in their practice, including on a regular basis.

The final question aimed to reassess the respondent’s opinion after completing a series of questions related to remote medical services and to provide an overall evaluation of their attitude.

**Figure 6:**
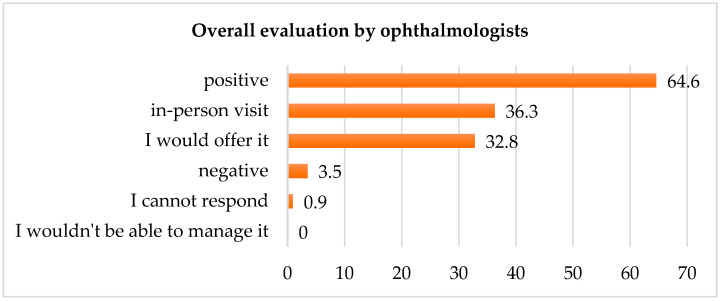
Ophthalmologists’ opinions regarding telemedicine services (in %). Multiple responses were allowed. Percentages were calculated based on the total number of respondents (n = 113).

As respondents were allowed to select more than one answer, the total number of responses was 156. The results showed that the majority of participants (64.6%, n = 73) had a positive attitude toward digital medical services, and 32.8% (n = 37) stated that they were willing to actively offer them. Nevertheless, 36.3% (n = 41) preferred in-person examinations, underscoring the continued importance of traditional consultation methods. Negative attitudes were low (3.5%, n = 4), and none of the respondents believed that they would be unable to cope with digital technologies. The data are presented in Figure 6.

A total of 21 respondents provided open-ended comments. These comments were reviewed and grouped into broad descriptive categories reflecting common themes expressed by participants. The purpose of this summary was to provide contextual insights into the quantitative findings rather than to perform a formal qualitative content analysis. Overall, the comments reflected predominantly positive attitudes toward teleophthalmology, mobile applications, and artificial intelligence. Respondents indicated support for the implementation of teleophthalmology, mobile applications, and AI, which were perceived as tools for improving the quality of care and optimizing workflow. The need for a balanced application of technology was emphasized, with telemedicine viewed primarily as suitable for consultations and follow-up of patients who previously underwent an in-person examination. The findings indicated that AI was perceived mainly as a supportive tool for automating administrative and documentation tasks, while its use in clinical decision-making was approached with greater caution. Participants expressed interest in digital forms of training and highlighted the importance of gradual and controlled implementation of technological innovations, in order to avoid additional professional burden.

## 4. Discussion

The results of the present study indicate that teleophthalmology services have not yet become routine practice among ophthalmologists; however, attitudes toward them were generally positive and reflect readiness for future development. More than one third of the surveyed physicians (35.4%) reported that they had already provided telemedicine services, and an additional 19.5% stated that they planned to implement them in the future. These findings suggest a gradual integration of telemedicine into ophthalmological practice, albeit at a moderate pace.

Similar trends are observed internationally. International studies conducted before and during the COVID-19 pandemic have demonstrated increasing institutional interest in telemedicine implementation among healthcare providers and healthcare organizations [17]. The lower proportion reported in the present study likely reflects specific national characteristics, including a more limited regulatory framework, the absence of sustainable financing models, and less developed telemedicine infrastructure in Bulgaria.

Data from India during the COVID-19 pandemic further confirmed the potential of teleophthalmology. Studies conducted during the COVID-19 pandemic in India reported generally positive experiences and satisfactory acceptance of virtual ophthalmology services among ophthalmologists [18]. In the present study, a similarly high level of satisfaction was observed among physicians who had already used teleophthalmology services—over 70% rated their experience as good or excellent. This suggests that once practical experience is gained, attitudes toward remote consultations tend to become more positive.

Nevertheless, both international findings and the results of the present study reveal substantial reservations. Published reviews suggest that although ophthalmologists demonstrate willingness to participate in telemedicine services, concerns regarding diagnostic confidence and remote clinical decision-making remain substantial [19]. Similarly, in the present study, attitudes toward remote treatment recommendations and emergency consultations were more cautious, with this hesitancy being particularly pronounced in the context of glaucoma. This may be explained by the specific nature of the disease, which requires regular instrumental examinations such as intraocular pressure measurement, optical coherence tomography, and visual field testing—methods that are difficult to apply reliably in a remote setting.

Particularly indicative are the findings related to the influence of prior knowledge of the patient. In the present study, ophthalmologists demonstrated a clearly more positive attitude toward remote consultations with patients they already knew, compared to entirely unfamiliar individuals. This underscores the importance of prior clinical contact, available medical documentation, and established trust, which are essential for the quality of remote communication and the safety of clinical decision-making.

This observation is consistent with international publications suggesting that telemedicine consultations are most appropriate for the follow-up of previously diagnosed and known patients, particularly in chronic conditions requiring long-term monitoring.

The standards of the Royal College of Ophthalmologists in the United Kingdom for virtual glaucoma clinics further emphasize that remote models of care are most effective for risk-stratified patients with an established diagnosis, whereas primary diagnosis and emergency cases should remain within the scope of in-person care [14].

Data from pilot studies and national surveys in the United Kingdom indicates good acceptability of virtual clinics for the follow-up of stable patients, contributing to resource optimization and reduced waiting times [15].

Regarding AI, the results of the present study demonstrated more moderate attitudes and limited practical use. This contrasts with data from a February 2025 survey by the American Medical Association, according to which approximately two out of three physicians (around 66%) use AI in their practice, reflecting a 78% increase compared to 2023 [20]. In the present study, more than half of the surveyed ophthalmologists had never used AI, and only a small proportion reported using it regularly. This difference likely reflects disparities in access to technology, the educational environment, and the level of institutional support.

Recent studies have demonstrated expanding applications of artificial intelligence in glaucoma care, including early diagnosis, progression prediction, and integration of multimodal imaging data [21]. Recent evidence also suggests that AI-assisted systems may improve diagnostic accuracy and support clinical decision-making in glaucoma management [22]. AI-assisted teleophthalmology models may additionally improve access to ophthalmic services, particularly in underserved and remote regions [23]. Nevertheless, concerns regarding regulation, trust, and implementation remain important barriers to broader adoption. Recent systematic reviews have additionally highlighted the growing role of AI in predicting glaucoma progression and supporting long-term disease monitoring [24].

The use of solutions developed by OpenAI, including ChatGPT, is described in the literature as a tool for improving physician–patient communication, summarizing medical information, and supporting clinical decision-making [25]. At the same time, international studies report significant concerns that patient interaction with AI may lead to misinformation and negatively affect their understanding of their health condition [26]. These concerns are also reflected in the findings of the present study, where a substantial proportion of ophthalmologists expressed uncertainty or skepticism regarding the role of AI in clinical practice.

Scientific publications indicate that AI chatbots can function as effective supportive tools in formulating differential diagnoses, planning treatment, and automating administrative tasks [26,27]. Of particular interest is a 2023 study in which ChatGPT-4 demonstrated diagnostic and therapeutic accuracy comparable to—or even exceeding—that of ophthalmologists with a subspecialty in glaucoma [28]. Despite these findings, the results of the present study showed that Bulgarian ophthalmologists perceive AI primarily as an assistive rather than an autonomous clinical tool, which aligns with the ethical and professional principles of medical practice.

Attitudes toward and the practical use of AI by ophthalmologists cannot be examined in isolation from the broader systemic context and the increasing burden on ophthalmic care, which further underscores the importance and necessity of implementing digital solutions and innovative technological approaches in clinical practice. In the United Kingdom, ophthalmology has become one of the busiest outpatient specialties within the National Health Service (NHS), reflecting the increasing burden of chronic eye diseases and population aging [29,30]. This trend is driven both by population aging and by the rising prevalence of chronic eye diseases such as glaucoma, diabetic retinopathy, and age-related macular degeneration, all of which require long-term and regular follow-up.

In the context of limited human resources and a growing number of patients, the traditional care model based exclusively on in-person examinations is becoming increasingly difficult to sustain. In this setting, the development of AI models such as RETFound and their integration into clinical practice demonstrate significant potential to transform ophthalmic care. These technologies offer opportunities for automated analysis of imaging studies, early disease detection, risk stratification, and optimization of follow-up processes, which could lead to more efficient use of available resources and improved clinical outcomes for patients.

Recent high-impact scientific publications have demonstrated the potential of foundational retinal models trained using self-supervised learning approaches. The RETFound model, published in Nature, showed that a single pretrained retinal model can detect multiple ophthalmic diseases with high diagnostic accuracy [31]. Earlier studies published in Nature Medicine and The Lancet Digital Health further validated the clinical applicability of deep learning systems in real-world screening programs and patient referral algorithms [32]. These findings underscore that AI is moving beyond the experimental phase and entering routine clinical practice.

Despite their clear advantages, the practical implementation of such AI solutions reveals substantial challenges. Among the most prominent are the lack of sustainable business models, uncertainties regarding reimbursement, and the absence of unified and clearly defined regulatory frameworks governing the clinical use of AI algorithms. In addition, important issues arise concerning liability in cases of diagnostic errors, the protection of personal data, and the integration of AI tools into existing clinical pathways and health information systems.

Therefore, although digital technologies and AI offer genuine opportunities to address the growing burden in ophthalmology, their effective and safe implementation requires a systemic, phased, and properly regulated approach. This entails not only technological advancement but also the adaptation of health policies, targeted training of medical professionals, and the active involvement of clinicians in the development and validation of new solutions, in order to preserve the quality of medical care and maintain the central role of the physician in clinical decision-making.

These reservations are particularly pronounced in chronic conditions requiring long-term instrumental monitoring, such as glaucoma. Glaucoma is a chronic, progressive disease that significantly affects patients’ quality of life and requires sustained cooperation between physicians and patients. Effective communication has a well-documented therapeutic impact and is associated with improved diagnostic accuracy, higher patient satisfaction, and better adherence to treatment [33]. In this context, the findings of the present study—according to which more than 80% of ophthalmologists believed that digital communication and increased patient awareness would improve adherence to therapy in glaucoma—are fully consistent with international evidence.

Among the main barriers to the implementation of telemedicine are the lack of trained personnel, insufficient digital literacy, and the complexity of operating specialized medical devices [34]. International experience suggests that these challenges can be addressed through the introduction of clear requirements for training and certification of healthcare professionals working in the field of telemedicine [35].

In this regard, the findings of the present study are particularly indicative—85% of the ophthalmologists surveyed expressed willingness to participate in training related to contemporary trends and the provision of remote medical services. International data show that telemedicine is increasingly being integrated into the curricula of medical universities [36]. In Bulgaria, initial steps have been taken in this direction, including the Bachelor’s program in ‘Telemedicine‘ at the Medical University—Pleven and the Master’s program in ‘Artificial Intelligence in Healthcare‘ at the Medical University—Varna [37]; however, there is still a lack of structured and targeted training in telemedicine and AI specifically designed for physicians across different specialties.

Prospective initiatives such as the virtual hospital Agent Hospital in China demonstrate the transformative potential of AI, telemedicine, and virtual reality for both medical practice and education [38,39]. Despite these impressive developments, the present study clearly indicates that the adoption of such technologies among ophthalmologists remains cautious and requires a phased, well-regulated, and ethically grounded approach—one that integrates technological innovation with clinical expertise, professional responsibility, and patient needs.

### 4.1. Limitations of the Study

The study was conducted through an electronic survey distributed using a voluntary response method. This approach implies potential for selection bias, as ophthalmologists with a particular interest in digital technologies or in the topic of telemedicine may have been more likely to participate in the study.

Although the sample included 10.5% of all licensed ophthalmologists in Bulgaria, representativeness with respect to demographic characteristics could not be fully assessed. Official national data on the sex distribution of ophthalmologists is not publicly available, which limits the possibility of direct comparison with the structure of the professional population.

The statistical analysis was primarily exploratory and based on bivariate associations. Because demographic variables such as age, years of professional experience, and educational level may be interrelated, the observed associations should be interpreted with caution. Future studies with larger sample sizes should incorporate multivariate regression analyses to better evaluate independent predictors of teleophthalmology and artificial intelligence adoption.

Teleglaucoma and structured models of teleophthalmic care have not yet been widely implemented within the Bulgarian healthcare system. Consequently, a substantial proportion of the responses reflect attitudes and perceptions rather than long-term practical experience.

The study had a cross-sectional design and did not allow for analysis of changes in attitudes over time or assessment of the impact of future regulatory developments, training initiatives, or technological advancements. The results reflect the specific regulatory and organizational context of the Bulgarian healthcare system.

### 4.2. Strengths of the Study

The present study represents the first systematic assessment of the attitudes of ophthalmologists in Bulgaria toward the application of teleglaucoma, digital communication, and the use of AI in clinical practice. It included 10.5% of all licensed ophthalmologists in the country, providing substantial coverage of the professional population.

The study simultaneously examined several interrelated aspects of digital transformation in ophthalmology—telemedicine services, virtual consultations, mobile applications, and AI—thereby enabling a comprehensive assessment of specialists’ readiness to adopt innovative solutions.

The use of a standardized questionnaire with clearly defined response scales, together with the application of appropriate statistical methods, enhanced the reliability of the findings. Furthermore, the analysis of associations between attitudes and factors such as age, length of professional experience, and educational level provided a more in-depth interpretation of the process of adopting digital technologies.

In the context of limited national data on the subject, the present study may serve as a foundation for future policies, regulatory decisions, and educational programs in the field of digital health and telemedicine in Bulgaria.

## 5. Conclusions

The present study outlines the current landscape of attitudes among ophthalmologists in Bulgaria toward teleglaucoma, digital communication, and the use of AI in clinical practice. The findings demonstrate generally positive acceptance of teleophthalmology as a complementary tool, particularly for the follow-up of previously known patients and for specialist collaboration in clinical decision-making.

At the same time, respondents expressed reservations regarding remote diagnosis, emergency care, and the autonomous use of AI in ophthalmology. The main barriers identified were the lack of a clear regulatory framework, concerns regarding diagnostic reliability, and the need for instrumental monitoring in glaucoma management.

The high willingness to participate in training and the positive evaluation of digital communication indicate favorable conditions for the gradual implementation of telemedicine solutions in Bulgarian ophthalmic practice. In the context of the increasing workload in ophthalmology and the chronic nature of glaucoma, the integration of teleglaucoma and AI-assisted digital tools represents a promising and sustainable direction for future healthcare delivery. Successful implementation will require regulatory support, targeted professional training, and continued physician oversight in clinical decision-making.

## Figures and Tables

**Table 1:** Demographic characteristics.

Demographic Characteristic	Ophthalmologists n (%)
*sex*	
male	31 (27.4)
female	82 (72.6)
*age*	
min	25 years
max	80 years
mean	47.07 years
*Professional experience*	
Up to 5 years	21 (18.6)
5–10 years	13 (11.5)
10–20 years	23 (20.4)
Over 20 years	56 (49.6)
*Level of education*	
Masters	96 (85.0)
Doctorate	17 (15.0)
*Acquired specialization in ophthalmology*	
Yes	97 (85.8)
No	16 (14.2)
Total Respondents	113

**Table 2:** Attitudes toward the provision of medical services remotely.

Qn	Questions	No		Somewhat No		Unable to Respond/Determine		Somewhat Yes		Yes		Total	
		n	%	n	%	n	%	n	%	n	%	n	%
Q1	Would you offer remote medical services for consultation in cases of suspected glaucoma and/or for individuals belonging to risk groups for developing glaucoma?	13	11.5	31	27.4	8	7.1	31	27.4	30	26.5	113	100.0
Q2	Would you offer remote medical services to provide treatment recommendations for a patient with established glaucoma?	11	9.7	41	36.3	10	8.8	32	28.3	19	16.8	113	100.0
Q3	Would you agree to consult a patient with suspected or established glaucoma, whom you do not know, by telephone or computer instead of in person?	25	22.1	42	37.2	6	5.3	29	25.7	11	9.7	113	100.0
Q4	Would you agree to consult a patient with suspected or established glaucoma, whom you already know, by telephone or computer instead of in person?	8	7.1	26	23.0	2	1.8	41	36.3	36	31.9	113	100.0
Q5	Would you agree to monitor a patient with glaucoma diagnosed during a physical examination through a remote service for follow-up visits instead of in person?	14	12.4	35	31.0	10	8.8	33	29.2	21	18.6	113	100.0
Q6	Would you agree to consult a patient with glaucoma through a remote service in an emergency instead of in person?	20	17.7	44	38.9	10	8.8	26	23.0	13	11.5	113	100.0
Q7	Would you participate in training related to contemporary trends and the provision of remote medical services?	5	4.4	6	5.3	6	5.3	40	35.4	56	49.6	113	100.0
Q8	Would you conduct a real-time remote consultation with another ophthalmologist regarding your patient while the patient is present in your office?	2	1.8	9	8.0	11	9.7	43	38.1	48	42.5	113	100.0
Q9	Do you believe that offering patients a real-time remote consultation service with another specialist would improve your work?	4	3.5	12	10.6	30	26.5	37	32.7	30	26.5	113	100.0
Q10	Would you conduct a consultation or secondary examination of a patient with glaucoma through a virtual clinic?	7	6.2	21	18.6	17	15.0	43	38.1	25	22.1	113	100.0
Q11	Adherence to therapy among patients with glaucoma would improve with increased opportunities for physician-patient communication, including through digital technologies.	5	4.4	8	7.1	10	8.8	45	39.8	45	39.8	113	100.0
Q12	Adherence to therapy among patients with glaucoma would improve with increased awareness of the disease, including through mobile applications.	2	1.8	6	5.3	6	5.3	42	37.2	57	50.4	113	100.0
Q13	Do you consider mobile applications in the field of ophthalmology to be useful?	2	1.8	5	4.4	14	12.4	63	55.8	29	25.7	113	100.0
Q14	Do you believe that artificial intelligence can help improve physician-patient interaction?	8	7.1	16	14.2	31	27.4	34	30.1	24	21.2	113	100.0
Q15	Would you use artificial intelligence (e.g., ChatGPT) in your work if you had the opportunity?	11	9.7	17	15.0	29	25.7	32	28.3	24	21.2	113	100.0

## Data Availability

The data presented in this study are available on request from the corresponding author due to ethical, legal, and confidentiality considerations related to human participants.

## Supplementary Materials

The following supporting information can be downloaded at: https://www.mdpi.com/article/10.3390/healthcare14121696/s1, Supplementary Material S1: Questionnaire.
