# Supplementary material for: Teleophthalmology and Teleglaucoma in Clinical Practice: Attitudes of Ophthalmologists in Bulgaria
Source: Healthcare (Basel). 2026 Jun 13;14(12):1696. doi: 10.3390/healthcare14121696 (PMC13300617; doi:10.3390/healthcare14121696)
Supplement: Supplementary file 1 [file healthcare-14-01696-s001.zip › healthcare-4205174-supplementary.pdf]

MEDICAL UNIVERSITY – PLOVDIV
FACULTY OF PUBLIC HEALTH

The purpose of this survey is to study public attitudes among ophthalmologists regarding the use of remote medical services for eye diseases.
The survey is anonymous and does not collect personal data. The results obtained will be used solely for the purposes of the study.
Please respond by marking the chosen answer with an X in the circle. Only one answer is possible for each question unless otherwise stated. Complete with words where necessary.
If you are not a medical professional, please do not fill out this survey!

THANK YOU FOR YOUR ATTENTION AND TIME!

Questionnaire

Gender: ◯ Male ◯ Female Age /in completed years/................
Education: ◯ Master's degree ◯ Doctoral degree
Specialty in Ophthalmology: ◯ Yes ◯ No
Work experience as an ophthalmologist /in completed years/.......................
Do you live and work in Bulgaria: ◯ Yes ◯ No

1. Do you know what the term 'teleophthalmology' includes?
◯ Yes ◯ Rather yes ◯ Undecided ◯ Rather no ◯ No

2. Have you offered teleophthalmology services to your patients?
◯ Yes ◯ No, but I plan to offer them ◯ I am not familiar with this type of service
◯ No

If the answer is 'yes', go to other questions and then continue from question 3:
2-1 What do you offer remote medical services for?
◯ for consultation; ◯ for information only; ◯ for a second opinion; ◯ for regimen recommendation; ◯ for treatment; ◯ in case of emergency; ◯ for other /please specify/……………………………..

2-2 How do you conduct telemedicine consultations?

◯ by phone; ◯ via Skype/Viber/Messenger; ◯ via email; ◯ via specialized software; ◯ via virtual clinic; ◯ via mobile application; ◯ other

2-3 What is your satisfaction with the conducted telemedicine consultations?

◯ excellent; ◯ good; ◯ satisfactory; ◯ I am not satisfied; ◯ I would not offer again; ◯ other

2-4 Do you know the patients with whom you conduct remote meetings?

◯ yes; ◯ no

2-5 Have you conducted remote consultations with patients with suspected glaucoma or glaucoma patients?

◯ yes; ◯ no

3. Would you offer remote medical services for consultation of a patient with suspected glaucoma and/or from risk groups for developing glaucoma?

◯ Yes ◯ Rather yes ◯ Undecided ◯ Rather no ◯ No

4. Would you offer remote medical services for treatment recommendations for a patient with established glaucoma?

◯ Yes ◯ Rather yes ◯ Undecided ◯ Rather no ◯ No

5. Would you agree to consult a patient with suspected glaucoma or an established disease whom you do not know, using a phone or computer, instead of seeing them in person?

◯ Yes ◯ Rather yes ◯ Undecided ◯ Rather no ◯ No

6. Would you agree to consult a patient with suspected glaucoma or an established disease whom you know, using a phone or computer, instead of seeing them in person?

◯ Yes ◯ Rather yes ◯ Undecided ◯ Rather no ◯ No

7. Would you agree to monitor a patient with glaucoma established during a physical examination through a remote access service for follow-up check-ups, instead of seeing them in person?

◯ Yes ◯ Rather yes ◯ Undecided ◯ Rather no ◯ No

8. Would you agree to consult a patient with established glaucoma through a remote access service in case of an emergency, instead of seeing them in person?

◯ Yes ◯ Rather yes ◯ Undecided ◯ Rather no ◯ No

9. Do you think that remote access medical services should have a lower price than a standard examination?

◯ Yes ◯ Rather yes ◯ Undecided ◯ Rather no ◯ No

10. Do you think that remote access medical services should be paid for by the health insurance fund rather than by the patient?

◯ Yes ◯ Rather yes ◯ Undecided ◯ Rather no ◯ No

11. What digital device do you use or would you use in the future in your work? /more than one answer is possible/

◯ computer ◯ laptop ◯ tablet ◯ smartphone ◯ smart watch ◯ other – please specify......................................

12. How many hours on average per day do you use digital devices? /mark the answer closest to you/

◯ under 2 ◯ from 2 to 4 ◯ from 4 to 8 ◯ over 8 ◯ I do not use every day

13. Which of the following would you use for remote patient consultation? /more than one answer is possible/

◯ phone call ◯ Skype/Viber/Messenger ◯ specialized software ◯ mobile application

◯ email ◯ virtual clinic ◯ other – please specify..

14. What are your concerns regarding issues related to offering remote medical services? /more than one answer is possible/

◯ moral-ethical ◯ legal regulation ◯ occurrence of technical problems

◯ the service carries risks ◯ financially ineffective service - I have no concerns

◯ misuse of personal information ◯ difficulties in use

◯ other – please specify...

15. What are your concerns when patients search for information on the internet regarding their eye health? /more than one answer is possible/

◯ possible start of self-treatment ◯ unclear source of information
◯ technical obstacles ◯ incorrectly presented information
◯ the patient has no way of knowing if it applies to them ◯ I have no concerns
◯ other – please specify..

16. Would you participate in training on modern trends and offering remote medical services?

◯ Yes ◯ Rather yes ◯ Undecided ◯ Rather no ◯ No

17. Do you deal with glaucoma patients?

◯ yes, among my main professional interests ◯ yes, along with other patients ◯ rather yes
◯ rather no ◯ No

18. Would you conduct a consultation with another ophthalmologist for your patient through a remote service in real-time while they are in your office?

◯ Yes ◯ Rather yes ◯ Undecided ◯ Rather no ◯ No

19. Do you think that if you offer patients a remote service for consultation with another specialist in real-time, it would improve your work?

◯ Yes ◯ Rather yes ◯ Undecided ◯ Rather no ◯ No

20. Where do you get information to update your knowledge of eye diseases?

◯ from printed scientific literature, textbooks; ◯ from subscription to scientific online publications via email; ◯ from scientific online publications; ◯ from printed publications of pharmaceutical companies; ◯ from websites of pharmaceutical companies; ◯ from colleagues; ◯ from popular science sources; ◯ from websites of other eye practices;
◯ from congresses and conferences; ◯ from company symposia; ◯ from virtual webinars;
◯ other..; ◯ I know enough

21. Given your practice, do you consider it useful to add any of the following in communication with patients? /more than one answer is possible/

◯ real-time video call ◯ phone call ◯ email correspondence ◯ text messages /SMS/
◯ images /e.g., photos/ sent electronically ◯ virtual office with video call option
◯ online sharing of materials related to the patient's condition
◯ other………………………………………………………………………………. ◯ No

22. Would you conduct a consultation or a follow-up examination of a glaucoma patient through a virtual office?

◯ Yes ◯ Rather yes ◯ Undecided ◯ Rather no ◯ No

23. Do you consider mobile applications in the field of ophthalmology to be useful?

◯ yes, I use such ◯ yes, I don't use them but I approve of the idea ◯ Undecided ◯ rather no ◯ No

24. Do you think that glaucoma patients' adherence to therapy will improve if opportunities for doctor-patient communication are increased, including through the use of digital technologies?

◯ Yes ◯ Rather yes ◯ Undecided ◯ Rather no ◯ No

25. Do you think that glaucoma patients' adherence to therapy will improve if their awareness of the disease is increased, including through the use of mobile applications?

◯ Yes ◯ Rather yes ◯ Undecided ◯ Rather no ◯ No

26. Do you think that artificial intelligence can help in doctor-patient interaction?

◯ Yes ◯ Rather yes ◯ Undecided ◯ Rather no ◯ No

27. Would you use artificial intelligence /e.g., chatGPT/ in your work if you had the opportunity?

◯ Yes ◯ Rather yes ◯ Undecided ◯ Rather no ◯ No

28. Have you contacted artificial intelligence /e.g., chatGPT/ to ask questions related to your work?

◯ yes, regularly ◯ yes ◯ I am not familiar ◯ no ◯ I would not do it

29. What is your general attitude towards using medical services via a digital device – for information and/or remote consultation? /more than one answer is possible/

◯ positive ◯ I would offer ◯ I would not manage ◯ I prefer a personal visit
◯ negative ◯ Undecided ◯ other – please specify..

We would be grateful if you could share your opinion or recommendations regarding this survey:

....
